# Spermatozoa Transcriptional Response and Alterations in PL Proteins Properties after Exposure of *Mytilus galloprovincialis* to Mercury

**DOI:** 10.3390/ijms22041618

**Published:** 2021-02-05

**Authors:** Gennaro Lettieri, Rosaria Notariale, Alessia Ambrosino, Alfredo Di Bonito, Antonella Giarra, Marco Trifuoggi, Caterina Manna, Marina Piscopo

**Affiliations:** 1 Department of Biology, University of Naples Federico II, Via Cinthia, 21, 80126 Naples, Italy; gennarole@outlook.com (G.L.); alessia.ambrosino@gmail.com (A.A.); alfredo.db@hotmail.it (A.D.B.); 2 Department of Precision Medicine, School of Medicine, University of Campania “Luigi Vanvitelli”, Via Luigi de Crecchio, 80138 Naples, Italy; notarialer@gmail.com (R.N.); caterina.manna@unicampania.it (C.M.); 3 Department of Chemical Sciences, University of Naples Federico II, Via Cinthia, 21, 80126 Naples, Italy; antonella.giarra@unina.it (A.G.); marco.trifuoggi@unina.it (M.T.)

**Keywords:** mussel, HgCl_2_, sperm nuclear basic proteins, sperm chromatin, male gametes, reproduction

## Abstract

Mercury (Hg) is an environmental pollutant that impacts human and ecosystem health. In our previous works, we reported alterations in the properties of *Mytilus galloprovincialis* protamine-like (PL) proteins after 24 h of exposure to subtoxic doses of toxic metals such as copper and cadmium. The present work aims to assess the effects of 24 h of exposure to 1, 10, and 100 pM HgCl_2_ on spermatozoa and PL proteins of *Mytilus galloprovincialis*. Inductively coupled plasma–mass spectrometry indicated accumulation of this metal in the gonads of exposed mussels. Further, RT-qPCR analyses showed altered expression levels of spermatozoa *mt10* and *hsp70* genes. In *Mytilus galloprovincialis*, PL proteins represent the major basic component of sperm chromatin. These proteins, following exposure of mussels to HgCl_2_, appeared, by SDS-PAGE, partly as aggregates and showed a decreased DNA-binding capacity that rendered them unable to prevent DNA damage, in the presence of CuCl_2_ and H_2_O_2_. These results demonstrate that even these doses of HgCl_2_ exposure could affect the properties of PL proteins and result in adverse effects on the reproductive system of this organism. These analyses could be useful in developing rapid and efficient chromatin-based genotoxicity assays for pollution biomonitoring programs.

## 1. Introduction

In recent decades, large quantities of pollutants have been released into the marine environment and estuaries [1]. Of all the pollutants, toxic metals have long been considered the main contaminants in the marine environment, representing a serious danger to marine organisms [2,3,4]. In marine environments, due to their benthic and sedentary way of life, bivalves are easily exposed to environmental pollution (toxic metals, persistent organic pollutants, etc.) and bioaccumulate these toxicants [5]. For this reason, these organisms are typically used as models in the field of environmental toxicology [6,7]. In particular, the mussel *Mytilus galloprovincialis* has been identified by several authors as a bioindicator that responds quickly to environmental pollution, with a wide spatial distribution and economic relevance. In the life cycle of bivalves, the early developmental stages result in being most susceptible to various pollutants, such as toxic metals [8], pesticides [9], and antifouling paints [10], and it has been demonstrated that the concentrations of toxic metals able to cause lethal toxicity in embryos and larvae are much lower than those that are lethal to adults [11,12]. Among toxic metals, mercury (Hg) is one of the most toxic nonessential metals [13]. Mercury contamination in seawater is an issue to the environment and human health. In fact, concentrations of Hg species in seawater are very low, about 1 pM [14], but are sufficient to drive bio-accumulation and -magnification of this toxic metal to levels in marine organisms that can pose human and ecological health risks [15,16]. In addition, Hg has also resulted in being more toxic in comparison to other metals, such as lead (Pb) and cadmium (Cd), to *M. galloprovincialis* embryos [17]. The toxicity of mercury has been evaluated at high doses (micromolar), in some organisms, such as the European clam (*Ruditapes decussatus*), in which Hg significantly reduced sperm viability [18] and also in *Ciona intestinalis* on which mercury affected early developmental stages. In addition, this metal impaired the testes function of the tropical fish *Gymnotus carapo* (L.), producing a decrease in sperm count and the alteration in sperm morphology [19]. Moreover, an in vivo study performed by Lahnsteiner et al. (2004) [20] showed that mercury had significantly decreased the percentage of sperm motility and velocity of *Clarias gariepinus* and *Lota lota* [20]. Further, early transcriptional changes induced in vivo in the mussel gills by a combination of nanomolar concentration of Cd, Cu, and Hg were also reported [21]. Differential toxicity has also been observed among species. In particular, the embryos and larvae of *Crassostrea gigas* were more sensitive to copper, yet *Paracentrotus lividus* embryos and larvae were more sensitive to lead and mercury [22]. Finally, Beiras et al. [23] exposed different life stages of *M. galloprovincialis* to mercury and reported a consistent decreased sensitivity to the metal as developmental stages increased in age. To the best of our knowledge, the literature lacks information regarding the effects of mercury concentrations, similar to those present in the waters of the Mediterranean basin and the North Atlantic oceans, on *M. galloprovincialis* sperm nuclear basic proteins (SNBP) and DNA. To this aim, in the present work, we exposed *M. galloprovincialis* for 24 h to three picomolar doses (1, 10, and 100 pM) of HgCl_2_. After exposures, we measured the accumulation of mercury in the gonads of exposed mussels and the expression of the stress genes *mt10* and *hsp70* in spermatozoa. Further, we analyzed the possible changes in the electrophoretic pattern and in the DNA binding ability of protamine-like (PL) proteins. Finally, we analyzed the ability of PL proteins to protect DNA from the action of free radicals, as sperm DNA fragmentation is one of the main causes of infertility [24].

## 2. Results

### 2.1. Evaluation of Accumulation of Mercury in Male Gonads

**Figure 1:**
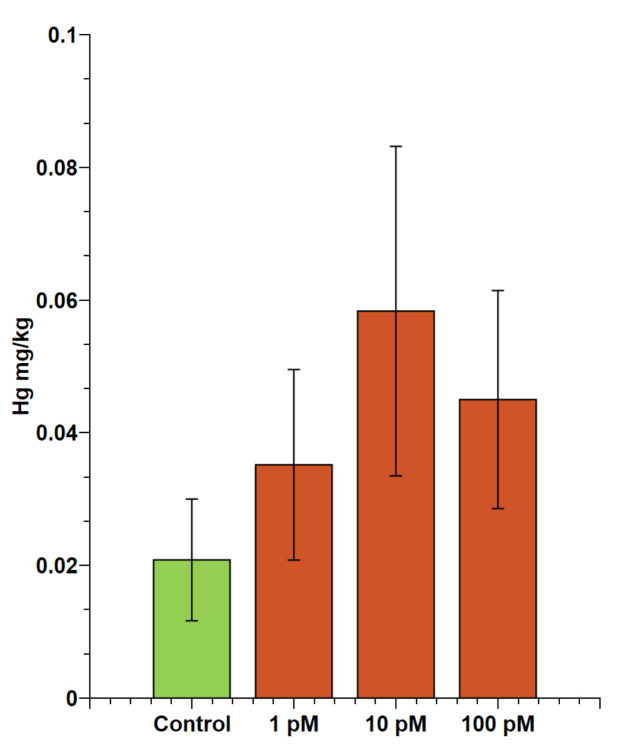
ICP-MS analyses of mercury bioaccumulation in gonads of *M. galloprovincialis*. Mercury concentration was evaluated after 24 h of exposure of mussels to 1, 10, and 100 pM HgCl_2_ in laboratory tanks. The values are expressed based on the wet weight basis. All values represent the mean ± S.D. obtained from 6 gonads of mussels from the same tank.

The possible accumulation of mercury in the gonads of male mussels exposed to HgCl_2_ was evaluated using inductively coupled plasma–mass spectrometry (ICP-MS). These analyses showed mercury accumulation in gonads after exposure for 24 h to 1, 10 and 100 pM HgCl_2_. In particular, the amount of mercury found in the gonads of unexposed mussels was 0.02 ± 0.01 mg/kg. As regards the exposed mussels, the values were: 0.04 ± 0.01 mg/kg for 1 pM; 0.05 ± 0.03 mg/kg for 10 pM; and 0.04 ± 0.02 mg/kg for 100 pM (Figure 1).

### 2.2. Spermatozoa Gene Expression

**Figure 2:**
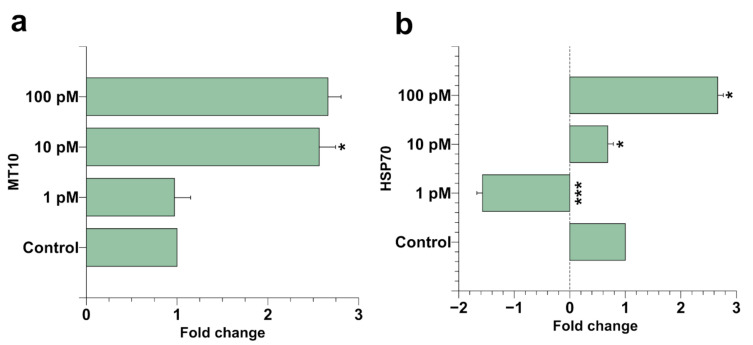
RT-qPCR expression analysis of *M. galloprovincialis* spermatozoa *mt10* and *hsp70*. In the figure, the fold change of the expression of the transcripts of *mt10* (**a**) and *hsp70* (**b**) is reported in the three HgCl_2_ exposure conditions with respect to the control condition (unexposed mussels), after determining their relative expression in comparison to the reference housekeeping 18S gene. Mussels were unexposed and exposed to 1, 10, and 100 pM HgCl_2_. Values are presented as mean ± S.D. (*n* = 6); asterisks indicate a statistically significant difference compared to unexposed mussels: * = *p* < 0.05; *** = *p* < 0.001.

After mussel exposure, a possible stress at the *M. galloprovincialis* spermatozoa level was assessed by quantitative reverse transcription polymerase chain reaction (RT-qPCR). For this aim, we measured the level of the stress genes *hsp70* and *mt10* in spermatozoa of mussels exposed to the three different concentrations of HgCl_2_ (1, 10, and 100 pM). It was found that *mt10* was on the order of about threefold over the control after 10 and 100 pM HgCl_2_ exposure. *hsp70* was hyper-expressed at the same extent after 100 pM HgCl_2_ exposure, while after 10 and 1 pM HgCl_2_ exposure, this gene resulted in being hypo-expressed, particularly at 1 pM HgCl_2_. In this latter condition, the hypo-expression of this gene was about 1.5 times compared with the control condition. These results showed that the exposure of mussels to these HgCl_2_ doses produced a stress condition for *M. galloprovincialis* spermatozoa (Figure 2).

### 2.3. Acid-Urea (AU)-PAGE of M. galloprovincialis PL-Proteins

**Figure 3:**
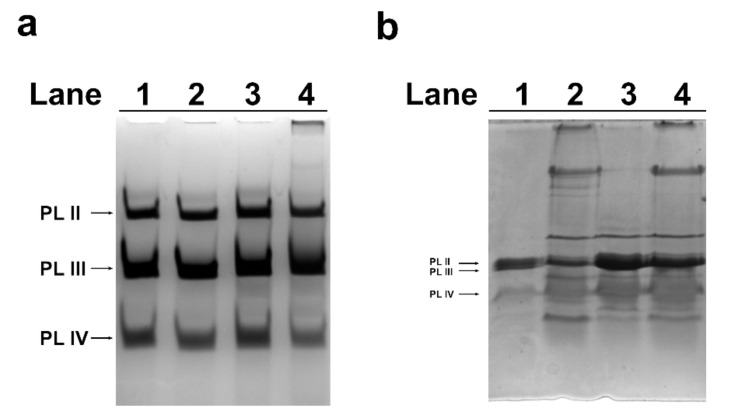
Electrophoretic analyses by acid-urea (AU)-PAGE (**a**) and SDS PAGE (**b**) of *M. galloprovincialis* protamine-like (PL) proteins extracted from unexposed (lane 1) and exposed mussels (lanes 2, 3, and 4): To 1, 10, and 100 pM HgCl_2_, respectively.

The alteration of *hsp70* and *mt10* genes expression in spermatozoa was indicative of a stress condition after mussels exposure to HgCl_2_. For this reason, the electrophoretic pattern of PL proteins extracted from mussels exposed to the three different concentrations of HgCl_2_ was determined by acetic acid-urea polyacrylamide gel electrophoresis (AU-PAGE). No significant differences were found in the electrophoretic pattern of PL proteins extracted from mussels exposed to the three different conditions compared with unexposed mussels (Figure 3a). By contrast, differences were found by analyzing the same proteins by SDS-PAGE (Figure 3b). As shown in this figure, the samples of PL proteins extracted from exposed mussels (lanes 2–4) presented, especially at 1 and 100 pM (Figure 3b, lane 2 and 4), additional protein bands with reduced mobility compared to control PL, likely indicative of the formation of several protein aggregates.

### 2.4. Electrophoretic Mobility Shift Assay (EMSA) Assay

**Figure 4:**
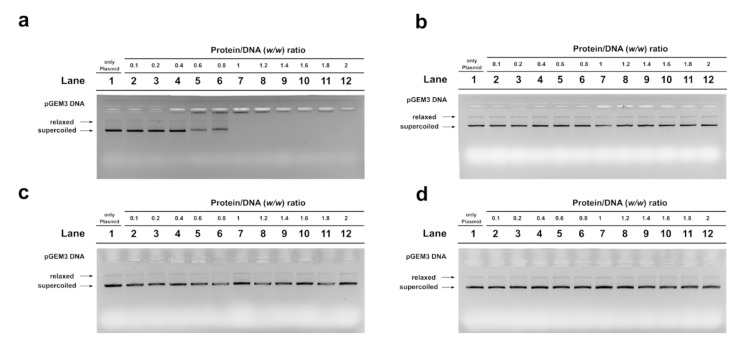
Electrophoretic mobility shift assay (EMSA) analyses of DNA binding ability of PL proteins from unexposed (**a**) and 1, 10, and 100 pM HgCl_2_-exposed mussels (**b**–**d**), using plasmid DNA. Numbers on the wells indicate the PL protein/DNA (*w*/*w*) ratios used; only plasmid denotes pGEM3 plasmid DNA with no proteins added.

Because several differences were found in the SDS-PAGE analysis of PL proteins extracted from mussels exposed to the three different concentrations of HgCl_2_, the DNA binding affinity of these PL proteins was investigated. For this aim, electrophoretic mobility shift assay (EMSA) was performed, using pGEM3 plasmid DNA as a probe, under the same conditions described in Vassalli et al. (2015) [25]. It was referred to as “DNA saturation” when all the plasmid DNA was close to the well. In the control condition (unexposed mussels), the DNA saturation was achieved at a PL proteins/DNA ratio of 1.0 (Figure 4a, lane 7), while at 1, 10, and 100 pM HgCl_2_ conditions, DNA saturation was not observed until a PL proteins/DNA ratio of 2 (Figure 4b–d, lanes 12). These results indicated a lower DNA binding ability of the PL proteins extracted from exposed mussels with respect to those obtained from control mussels.

### 2.5. DNA Protection Analysis

**Figure 5:**
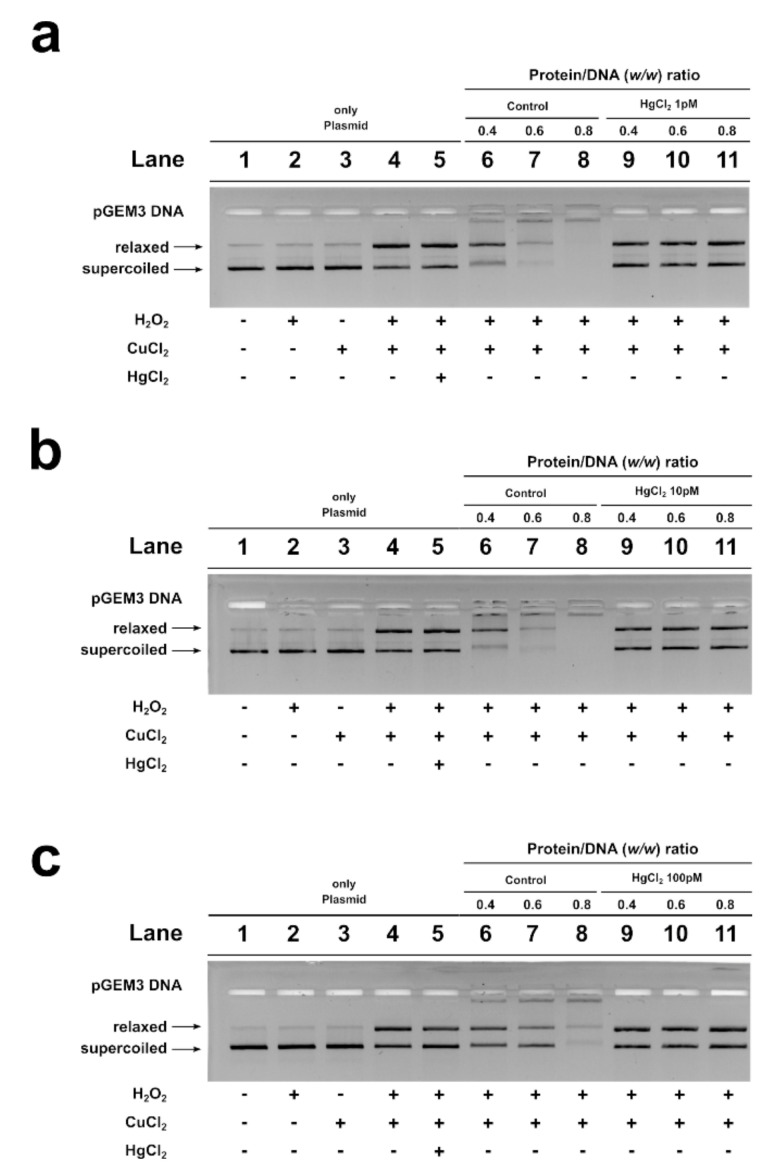
DNA protection analysis on 1% agarose gel of pGEM3 plasmid DNA in the presence of increasing (0.4, 0.6, and 0.8) PL/DNA ratios. Control (lanes 6, 7, and 8 of all the panels); 1 pM HgCl_2_ (lanes 9, 10, and 11 of panel (**a**)); 10 pM HgCl_2_ (lanes 9, 10, and 11 of panel (**b**)); 100 pM HgCl_2_ (lanes 9, 10, and 11 of panel (**c**)).

An assay was also performed to determine the potential of *M. galloprovincialis* PL proteins to protect DNA from oxidative damage. A condition was created in which plasmid DNA damage occurred. In this condition, the plasmid DNA was placed in the presence of 10 µM H_2_O_2_ and 5 µM CuCl_2_ in order to cause the Fenton reaction and produce DNA breakage. The result obtained under this condition is shown in lane 4 of all agarose gels in Figure 5, and it can be seen that more than 50% of the plasmid DNA was in the relaxed form. The addition of PL proteins from unexposed and exposed mussels to this mixture, in protein/DNA ratios of 0.4, 0.6, and 0.8, produced different effects. In particular, when PL proteins from unexposed mussels were added, already at a protein/DNA ratio of 0.4, the entity of DNA breakage was lower with respect to the damage condition (compare lanes 6 and 4 in Figure 5). At 0.6 and 0.8 protein/DNA ratios, DNA damage was not observed, suggesting that these PL proteins produced complexes capable of protecting DNA. In fact, in this latter case, at increasing PL proteins/DNA ratios, the plasmid DNA bands corresponding to supercoiled and relaxed forms became less intense as DNA saturation took place, detectable by the appearance of a high-molecular-weight DNA band close to the well. The same assays performed by using the PL proteins from exposed mussels produced different results. In fact, the PL proteins obtained from mussels exposed to all HgCl_2_ doses were unable to produce complexes that protected DNA from oxidative damage (lanes 9–11 of panels a (1 pM), b (10 pM), and c (100 pM)).

## 3. Discussion

Some metals are important to maintain several biochemical and physiological functions in living organisms when they are in very low concentrations; however, they become toxic when they exceed certain threshold concentrations and represent a problem of increasing relevance for ecological, evolutionary, nutritional, and environmental reasons [26,27,28,29,30].

Human industrial activities have caused an increase in levels of Hg in the air, soil, and fresh and sea waters, and bioaccumulation along the food chain. Given the widespread exposure of organisms and the well-known toxicity of this metal, there is growing concern that exposure to mercury also at low levels may have many different adverse effects on different biological functions, including reproduction. In our previous works, we have reported alterations in the properties of *M. galloprovincialis* PL proteins after 24 h of exposure to subtoxic doses of toxic metals such as copper and cadmium [31,32]. Thus, in this work, we have evaluated the effects on the reproductive health of male *M. galloprovincialis* after exposure to mercury doses similar to those present in the waters of the Mediterranean basin and the North Atlantic oceans, sites where metal pollution has previously been reported [14,33,34].

For this aim, we exposed mussels for 24 h in laboratory tanks to 1, 10, and 100 pM HgCl_2_ and measured the mercury accumulation and the *mt10* and *hsp70* expression, in the gonads and spermatozoa of exposed mussels, respectively. This is because, in our previous studies, it has been demonstrated that mussel gonad has the same accumulation capacity as gill, at least for copper. In addition, this latter metal induces alterations in the expression level of *mt10* in spermatozoa [31]. As reported by several authors [35,36], *Mytilus* species are able to synthesize the metal binding protein, metallothionein, for their detoxification.

In general, *mt10* genes appear to be highly expressed at baseline and can respond to both essential (Cu, Zn) and nonessential (Cd, Hg) toxic metals [37,38].

Indeed, our data are in accordance with the literature; the mussels showed higher expression levels of *mt10* after mercury contamination (Figure 2); in particular, the treatment of 100 pM showed an expression level increased about three times compared to the control condition (Figure 1). Expression levels of *hsp70* in mussel spermatozoa were also analyzed. HSP acts to prevent protein aggregation and to maintain functional conformations. Our RT-qPCR analysis showed significant and different responses in *hsp70* expression levels in mussel spermatozoa after the three HgCl_2_ tested exposure doses. In particular, the extremely low expression of hsp70 was found at the lowest concentration used (Figure 2b), suggesting an impairment of the biochemical mechanism underlying the heat shock response. Other studies have suggested that the downregulation of this gene is due to some factors that influence the stability and translation of mRNA [39].

Furthermore, even at the concentration of 10 pM of HgCl_2_, a slight downregulation of the *hsp70* gene was observed in the spermatozoa of mussels. To our knowledge, this is the first study demonstrating the downregulation of the *hsp70* gene in spermatozoa under metallic stress at low concentrations and within short acute exposure, which suggests a high sensitivity of spermatozoa in the response to mercury.

Interestingly, the increased HgCl_2_ concentration (100 pM) upregulated the transcription of the *hsp70* gene in mussel spermatozoa, differently to low concentrations (1–10 pM). This transcriptional regulation of *hsp70* could probably be needed to improve mercury tolerance, as high levels of HSP have been shown to protect against the negative impact of metals on protein integrity [40,41].

Considering the high efficiency of spermatozoa in the response to mercury, possible alterations in the properties of PL proteins, which represent the main component of the basic nuclear proteins that organize sperm DNA, were also evaluated. In particular, the electrophoretic pattern and DNA binding affinity of these proteins were analyzed after exposure of mussels to HgCl_2_. No significant differences in the electrophoretic pattern of PL proteins were found by AU-PAGE between unexposed and exposed mussels (Figure 3a). However, by SDS-PAGE, several protein bands with reduced mobility were found in the samples of PL proteins extracted from spermatozoa of mussels exposed to all the HgCl_2_ doses (Figure 3b). These protein bands could represent aggregates of PL proteins. Considering these alterations in PL proteins, their ability to bind DNA was tested. As a matter of fact, the decreased DNA binding capacity of PL proteins from exposed mussels was found for the pGEM3 DNA plasmid (Figure 4); in fact, DNA saturation was not achieved even at a proteins/DNA ratio of 2. Consistent with our previous work, it was confirmed by EMSA that the extracts of PL proteins (containing PL-II, PL-III, and PL-IV) from unexposed *M. galloprovincialis* interacted with DNA in “all or nothing” mode [4], as sperm H1 histones and *C. variopedatus* PL protein [42,43,44,45,46] and the DNA saturation were achieved at a proteins/DNA ratio of 1. The PL proteins obtained after all HgCl_2_ exposures maintained the same DNA binding mode of control PL proteins, differently from those obtained after copper mussels exposure [31]. In that case, we observed an increase in DNA binding affinity and also a change in DNA binding mode from “all or nothing” to “intermediate mode,” the typical DNA binding mode of somatic H1 histone [45]. These alterations could result in a change in the ability of PL proteins to bind and condense DNA and, in turn, abrogate their canonical role of DNA protection. In fact, in our previous studies, it has been reported that, in some cases, sperm nuclear basic proteins are involved in DNA oxidative damage [47].

Indeed, it was observed that all PL proteins extracted from spermatozoa of exposed mussels to mercury were unable to protect DNA from the action of copper and hydrogen peroxide (Figure 5). Taken together, our findings provide additional information that offers new insights into the mechanisms of mercury toxicity on the reproductive system of *M. galloprovincialis*. Additionally, PL protein studies could be useful for developing rapid and effective chromatin-based genotoxicity tests for biomonitoring programs for heavy metal impact assessment and species management. Therefore, further investigations are needed to understand the specific mechanisms of the action of mercury, leading to a more complete understanding of the mussel’s response to stress of this toxic metal.

## 4. Materials and Methods

### 4.1. Ethics Statement

This research was performed on the marine invertebrate *M. galloprovincialis* (Lamarck, 1819), which is not protected by any environmental agency in Italy. This study was conducted in strict accordance with European (Directive 2010/63) and Italian (Legislative Decree n. 116/1992) legislation on the care and use of animals for scientific purposes.

### 4.2. Evaluation of Accumulation of Mercury in Male Mussel Gonads

Mercury quantification was performed as follows: Samples consisting of single gonads of male mussels were digested by using 1 mL of ultrapure nitric acid (HNO_3_ ≥ 69%, *v*/*v*, Sigma Aldrich) in a microwave system equipped with an autosampler (CEM DISCOVER SP-D, CEM Srl, Cologno al Serio, Bergamo, Italy), according to the digestion procedure UNI EN 13805:2014 [48]. Samples were diluted to 10 mL with a solution of HNO_3_, 2%, *v*/*v*. For each digestion batch, a blank digestion of ultrapure nitric acid was performed to check for metal contamination, and a quality control shellfish-based sample (QMAS, Sample 741, LGC Standard) was analyzed to control the effectiveness of digestion.

The elemental analysis was conducted by inductively coupled plasma–mass spectrometry (ICP-MS, Aurora M90 Bruker, Billerica, MA, USA). Element concentrations were determined from a calibration curve calculated on the basis of five concentrations for the analyzed elements obtained from certified standard solutions. All the materials and reagents used were checked for metal contamination through repeat analysis of method blanks. Standard samples were processed at the beginning and every 10 samples in the analytical batch to verify the instrument calibration.

The accuracy of the method was evaluated by analyzing the CRM MT-742 of the fish-based sample from interlaboratory comparison (QMAS, Round 295, LGC Standard), obtaining a recovery value of 93%.

The analyses were performed on 6 samples of each type (control, exposed to 1 pM, exposed to 10 pM, and exposed to 100 pM HgCl_2_).

### 4.3. Mussels Sampling and HgCl_2_ Exposure

To investigate the specific effects of HgCl_2_, mixed-sex and medium-shell-size specimens of *M. galloprovincialis* were used (length 4.95 ± 0.17 cm), kindly provided by Eurofish Napoli S.R.L. Bacoli. The mussels were exposed to three concentrations of HgCl_2_ (1, 10, and 100 pM) as previously described for other toxic metals in Piscopo et al., 2016 [5], in laboratory plastic tanks (36 × 22 × 22 cm), each containing 6 L of 33‰ artificial sea water (ASW) with the following composition for 1 L: NaCl 29.2 g; KCl 0.60 g; MgCl_2_ 1.2 g; NaHCO_3_ 0.20 g; and CaCl_2_ 1.08 g. In particular, 13 mussels were placed in any tank for 24 h at 18 ± 1 °C. Water and metal salts were changed every 12 h during treatment. For each system, dissolved oxygen and temperature were recorded at predetermined time intervals. The experiments were performed in the winter period, January–February 2020. Tanks containing only ASW were used as a control for unexposed mussels. Two tanks were used for each condition for a total of eight tanks as already described in Lettieri et al., 2019 [49].

### 4.4. Spermatozoa Sampling and Processing

After 24 h of exposure to 1, 10, and 100 pM HgCl_2_, collection of spermatozoa from male mussels was performed. Mussels were then opened using a knife, taking care not to cut the soft tissues. Subsequently, after stimulating male gonads with a glass Pasteur pipette and the help of seawater, gametes were obtained and subjected to microscopic examination to identify the sex of the mussels and the sexual maturity on the basis of a morphological and seminal analysis as previously described in Piscopo et al., 2018 [3]. Spermatozoa were collected as reported in Vassalli et al., 2015 [25], with a glass Pasteur pipette. In brief, the semen collected from all the male mussels contained in the tanks corresponding to a specific µM HgCl_2_ condition were pooled and centrifuged at 1000× *g* for 2 min at 4 °C in order to remove the debris. To collect the spermatozoa, the supernatant obtained was centrifuged at 9000× *g* for 10 min at 4 °C. Pellets containing spermatozoa of about 200 mg were recovered and stored at −80 °C for the further investigations.

### 4.5. RNA Extraction and RT-qPCR

Total RNA was purified from spermatozoa of mussels unexposed (control) and exposed to the three different concentrations of HgCl_2_ by using the Trizol reagent (Invitrogen, Carlsbad, CA, USA) according to the manufacturer’s instructions. The quantification and quality of RNA samples were controlled with a UV-Vis spectrophotometer (NanoDropH ND-1000, Waltham, MA, USA) and by 1% agarose gel electrophoretic analysis under denaturing conditions [50]. Equal amounts of RNA obtained from the spermatozoa of mussels unexposed and exposed to HgCl_2_ were used in qPCR analyses. We used the procedure described in Basile et al., 2017 [26] with few modifications. First, we purified the RNA samples from genomic DNA with an Ambion (Austin, TX, USA) DNA-free kit and then performed the retrotranscription with M-MLV reverse transcriptase (ImpProm II kit, Promega, Madison, WIS, USA). Then, 1 μg of RNA from each condition was used to perform cDNA syntheses using random hexamers (0.5 µg/µg RNA). For the determination of genes expression by real-time PCR, 100 ng of the cDNA was used and 10 μM of each forward and reverse primers was used in a final volume of 50 μL using SYBR Green PCR Master Mix Kit (Applied Biosystems, Foster City, CA, USA) with the 7500 Real Time PCR System (Applied Biosystems, Foster City, CA, USA). The conditions of qPCR were as follows: All PCR reactions were performed for 40 cycles with the following specifications: Denaturation at 95 °C for 15 min; annealing and elongation at 60 °C for 1 min. The primer set used was designed using the open-source software Primer3, starting from the sequences indicated with the accession numbers reported in the last column of the table shown in Table 1. Before real-time PCR, it was verified by PCR that each primer pair produced a single primary amplicon. qPCR product dissociation curves for all transcripts gave single peaks.

The results were analyzed using the ViiA™-7 Software (Foster City, CA, USA) and exported into Microsoft Excel (Redmond, WA, USA, ver. 2009—build 13231.20262). To determine relative gene expression values, the ΔΔCt method was used [51]. All samples were processed with technical triplicates. Data for each gene were normalized against 18S ribosomal RNA. The expression levels of this gene were essentially stable as also reported by other authors [38]. The change in expression of *mt10* and *hsp70* transcripts related to reference 18S rRNA in the spermatozoa samples from mussels exposed to HgCl_2_ compared with control mussels was measured.

### 4.6. PL Proteins from M. galloprovincialis Spermatozoa Extraction and Analyses

Extraction of protamine-like proteins from spermatozoa was performed using 5% perchloric acid (PCA) as previously described in [52]. For this work, we used *n* = 10 spermatozoa pellets deriving from each tank corresponding to a specific µM HgCl_2_ condition. Spermatozoa pellets were homogenized in a potter with 15 mL of distilled water, and then PCA was added. Acid extraction was performed as described by Vassalli and coworkers [25], and at the end of procedure, we extensively dialyzed the samples containing PCA-soluble PL-proteins against distilled water, in order to guarantee all PCA was removed. Finally, the extracted proteins were lyophilized and stored at −80 °C.

PL proteins were analyzed by AU-PAGE as previously described in Piscopo et al., 2018 [53]. Gels were then acquired using a Gel-Doc system (BioRad, Hercules, CA, USA) via Quantity One v.4.4.0 (BioRad, Hercules, CA, USA) software. The software ImageJ ver 1.50d (https://imagej.nih.gov/ij/), supported by the National Institute of Health (Wayne Rasband, National Institute of Mental Health, Bethesda, MD, USA), was used for the densitometric analysis of the protein bands on gel.

SDS-PAGE of PL proteins was performed as previously described in De Guglielmo et al., 2019 [32] with a few modifications. In particular, the stacking gel was constituted by 5.0% (*w*/*v*) acrylamide (acrylamide/bis-acrylamide 30:0.15) and the separating gel was at 18.0% (*w*/*v*) acrylamide (acrylamide/bis-acrylamide 30:0.15). At the end of the run, the gels were stained with Coomassie Brilliant Blue and acquired using a GelDoc system via Quantity One v.4.4.0 software (BioRad, Hercules, CA, USA). The densitometric analysis of gel bands was carried out using the software ImageJ.

### 4.7. Plasmid DNA Preparation and Analysis

pGEM3 plasmid DNA was prepared according to Carbone and coworkers [54]. The quantification and quality of plasmid DNA were conducted with a UV-Vis spectrophotometer (NanoDropH ND-1000, Waltham, MA, USA) and the integrity of DNA was analyzed by gel electrophoresis on 1% agarose gels in 89 mM Tris-HCl pH 8.0, 2 mM EDTA, and 89 mM boric acid (TBE).

### 4.8. Analysis of the Effect of M. galloprovincialis PL Proteins on DNA Electrophoretic Mobility

The DNA binding affinity of PL proteins was evaluated by performing Electrophoretic Mobility Shift Assay (EMSA), as described in Fioretti et al., 2012 [42]. For this analysis, we used 150 ng of plasmid pGEM3 DNA in the circular form and increasing amounts of proteins, expressed as protein/DNA w/w ratios in a final volume of 30 µL. The protein/DNA *w*/*w* ratios used ranged from 0.1 to 2. The results obtained were analyzed on 1% agarose gels. On the wells of the gels shown in the results, the protein/DNA *w*/*w* ratios in the different conditions tested are reported. DNA migration was visualized by staining gels with ethidium bromide (2 µg/mL) after electrophoresis.

### 4.9. DNA Protection Analysis

The PL ability to protect DNA from oxidative damage in the presence of 10 µM H_2_O_2_ and 5 µM CuCl_2_ was performed by using 150 ng of plasmid DNA (pGEM3) and PL extracted from mussels unexposed and exposed to 1, 10, and 100 pM HgCl_2_. The conditions of DNA oxidative damage were redefined by using a fixed amount of H_2_O_2_ and increasing CuCl_2_ concentrations, in order to obtain more than 50% of the plasmid in the relaxed form, as shown in Figure S1. These conditions were obtained starting from those reported in Lettieri et al., 2020 [24]. The samples were prepared using the EMSA protocol described in paragraph 4.8. Plasmid DNA and proteins/DNA *w*/*w* ratios of 0.4, 0.6, and 0.8 were used. The interaction between DNA and PL proteins was for 5 min at room temperature, and then H_2_O_2_ and CuCl_2_ were added and the samples were incubated in the dark for 30 min at 37 °C. Just before electrophoresis analysis, in order to avoid the EDTA coordination of eventual metals, a sample buffer at 1X final concentration was added to the samples. The samples obtained were then analyzed on 1% agarose gel at 100 V for 30 min in TEB 1X, staining the gels with ethidium bromide (2 µg/mL) at the end. The images of the gels were acquired at the GelDoc Biorad (Hercules, CA, USA). All experiments were performed at least three times.

### 4.10. Statistical Analysis

Multiple group data were analyzed using one-way ANOVA and Dunnett’s test was used to compare means between the groups. Values were considered significant when *p* < 0.05. Statistically significant differences are defined at the 95% confidence interval. Data are shown as mean ± S.D. Analyses were made on the pool of spermatozoa collected from mussels of any different laboratory tank.

## Figures and Tables

**Table 1:** List of forward and reverse primers used for amplification of each gene analyzed and for the reference housekeeping 18S gene.

Gene	F-Primer	F-Primer Length	R-Primer	R-Primer Length	Accession Number
*18S*	GCCACACGAGATTGAGCAAT	20	CTCGCGCTTACTGGGAATTC	20	L244B9
*hsp70*	CGCGATGCCAAACTAGACAA	20	TCACCTGACAAAATGGCTGC	20	AY861684
*mt10*	GCCTGCACCTTGTAACTGTAT	21	CTGTACACCCTGCTTCACAC	20	AY566248

## Data Availability

Not applicable.

## Supplementary Materials

Supplementary Materials can be found at https://www.mdpi.com/1422-0067/22/4/1618/s1: Figure S1. Settings of the ideal conditions for pGEM3 plasmid DNA breakage in presence of CuCl_2_ and H_2_O_2_.

## Abbreviations

ASW	Artificial sea water
AU-PAGE	Acetic acid-urea polyacrylamide gel electrophoresis
SDS-PAGE	Sodium dodecyl sulphate–polyacrylamide gel electrophoresis
EDTA	Ethylenediaminetetraacetic acid
EMSA	Electrophoretic mobility shift assay
ICP-MS	Inductively coupled plasma–mass spectrometry
PCA	Perchloric acid
PL	Protamine-like
qPCR	Quantitative polymerase chain reaction
RT-qPCR	Reverse transcript quantitative polymerase chain reaction
SNBP	Sperm nuclear basic protein
