# Supplementary material for: Spermatozoa Transcriptional Response and Alterations in PL Proteins Properties after Exposure of Mytilus galloprovincialis to Mercury
Source: Int J Mol Sci. 2021 Feb 5;22(4):1618. doi: 10.3390/ijms22041618 (PMC7915165; doi:10.3390/ijms22041618)
Supplement: Supplementary file 1 [file ijms-22-01618-s001.pdf]

International Journal of
Molecular Sciences

MDPI

Supplementary Material

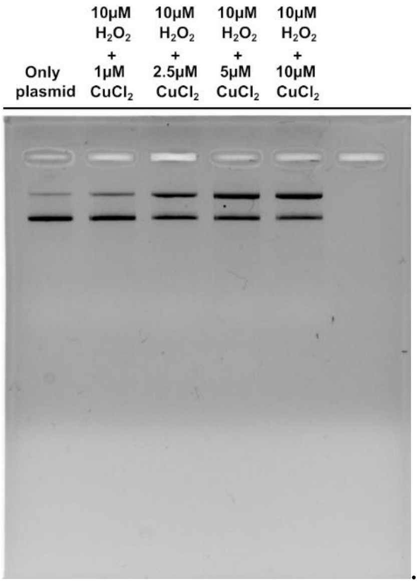

Figure S1. Settings of the ideal conditions for pGEM3 plasmid DNA breakage in presence of $CuCl_2$ and H_2O_2.
